# Traditional craftspeople are not copycats: Potter idiosyncrasies in vessel morphogenesis

**DOI:** 10.1371/journal.pone.0239362

**Published:** 2020-09-22

**Authors:** Enora Gandon, Tetsushi Nonaka, John A. Endler, Thelma Coyle, Reinoud J. Bootsma

**Affiliations:** 1 Institute of Archaeology, University College London, London, United Kingdom; 2 Graduate School of Human Development and Environment, Kobe University, Kobe, Japan; 3 Centre for Integrative Ecology, School of Life & Environmental Sciences, Deakin University, Waurn Ponds, Australia; 4 Institut des Sciences du Mouvement, Aix Marseille Université, CNRS, Marseille, France; University of Queensland, AUSTRALIA

## Abstract

Ceramics are quintessential indicators of human culture and its evolution across generations of social learners. Cultural transmission and evolution theory frequently emphasizes apprentices’ need for accurate imitation (high-fidelity copying) of their mentors’ actions. However, the ensuing prediction of standardized fashioning patterns within communities of practice has not been directly addressed in handicraft traditions such as pottery throwing. To fill this gap, we analysed variation in vessel morphogenesis amongst and within traditional potters from culturally different workshops producing for the same market. We demonstrate that, for each vessel type studied, individual potters reliably followed distinctive routes through morphological space towards a much-less-variable common final shape. Our results indicate that mastering the pottery handicraft does not result from accurately reproducing a particular model behaviour specific to the community’s cultural tradition. We provide evidence that, at the level of the elementary clay-deforming gestures, individual learning rather than simple imitation is required for the acquisition of a complex motor skill such as throwing pottery.

## Introduction

Social learning allows cultural traditions to persist over generations [1]. Among the processes underlying cultural transmission [2, 3], action imitation has been suggested as being particularly important and, perhaps, specific to humans [4, 5]. Indeed, accurate imitation of their accomplished elders’ way of doing, commonly denoted high-fidelity copying, is argued to allow learners to “ratchet up” existing knowledge, thereby reducing or eliminating the need to rediscover an effective way of solving each problem [6]. While presented as a general principle, to our knowledge the operation of high-fidelity copying has so far not been experimentally explored in behavioural traditions involving complex motor skills such as pottery and other handicrafts. Here we bring a motor behaviour perspective to bear on the question of whether potters, having learned the skill from elders within their community, could have done so by imitation. We do this by focussing on the observable results of potters’ clay-deforming technical gestures,

Pottery, the craft of making container objects out of clay, is an ancient human tradition [7]. Gradually superseding earlier coiling, the introduction of the throwing technique exploiting the fast-rotating wheel, dating back at least to the Middle Bronze II era (1750 BC), allowed substantial gains in production rate and product regularity [8, 9]. At the same time, however, it made the fashioning process considerably more difficult to master and thereby longer to learn [10, 11].

Starting from the moment the lump of clay is placed on the rotating wheel, the throwing process itself is characterized by an invariant sequence of general operations [9, 10, 12]. During the pre-forming phase the potter first centres the mass of clay on the wheel and subsequently sets the stage for the forming process by opening (hollowing) the centred lump of clay. During the forming phase thinning the clay walls brings out a preliminary roughout as the vessel rises from its base, while the final form is attained after the ultimate shaping operations. In fashioning the vessel, potters successively deploy several distinctive hand positions for contact with the clay [10]. Addressing the influence of cultural setting on wheel-throwing practices, in earlier work we analysed hand position sequences as observed in French, Indian and Nepalese potting communities [13–16].

Here we analysed the throwing process by tracking the potter-induced morphological changes in the clay body, from its initial pre-formed stage following centring and opening operations, up to the moment that the final form is reached. We emphasize that vessel morphogenesis, as studied here, allows capturing the essential result of each manual fashioning gesture in terms of the change in clay shape brought about by contact between the hands and clay. In so doing, it provides an integrative view of the potter’s shaping actions. Our analyses focused on variation in morphological development of the clay body, both amongst different potters and within individual potters, as they each repeatedly threw customary vessel types.

As a long-standing behavioural tradition, wheel-throwing clearly entails social learning and transmission [1, 17, 18]. Typical for such behavioural traditions, the seven Indian potters participating in the present study had learned the craft as apprentices guided by elder mentors who had previously learned it themselves in much the same way within the same tradition. If, like their elders, these potters had learned how to throw a particular type of vessel through high-fidelity copying, that is, by accurately imitating each step in their mentors’ way of doing [4–6, 19–21], within each community of practice [22] fashioning patterns would be culturally standardized. In the framework of the present study, such high-fidelity copying would therefore be expected to give rise to low variation among potters from the same community in the throwing process and, thereby, in the resulting final vessels forms.

Ethnoarchaeological studies examining the degree of product standardization (quantified by metric variability) in same-type vessel assemblages have pinpointed the essential role of economic specialization, with intensive production allowing potters to fully develop their skill. Highly standardized vessels can indeed only be consistently delivered by expert specialists, that is, by highly-skilled potters [23–25]. Becoming an expert in any area requires at least ten years of extended, deliberate practice [26] and genuinely mastering the craft of wheel-throwing is no exception to this rule [10]. The road to expertise is therefore long and demanding.

Rather than relying on invariably rehearsing the same gestures, deliberate practice has been argued to give rise to exploration and reorganizations of the skill [27, 28], so as to find suitable individualized solutions to the problems posed by the task at hand. While being capable of consistently obtaining the same desired result, experts thus typically reveal individual differences (‘styles’) between them in their way of doing [29–33]. For the present purposes, this inter-individual functional motor equivalence perspective [28, 34–36] suggests that the low variability that is expected in the final forms thrown by expert potters need not be associated with similarly low between-potter variability in the throwing process: different experts are likely to have found different routes to the same end result.

In the context of the present study, hypotheses about between-potter and within-potter variation in final vessel forms are therefore fairly clear-cut. We explore two alternative hypotheses: (1) if each potter learns by high-fidelity copying of a model, then throughout the forming process the degree of among-potter variation should be relatively small and of the same order as within-potter variation; (2) if each potter learns by deliberate practicing, then the degree of among-potter variation should be greater than within-potter variation and this pattern should be strongest at the earlier stages of morphogenesis, with among-potter variations decreasing as the vessel nears its final form. In order to test these hypotheses we requested potters to produce series of customary traditional forms, expected to yield final assemblages with low overall shape variability for each vessel type. Earlier experimental work [37, 38] indicated that for experts these final pottery assemblages would be characterized by low within-potter variability, resulting in subtle but discernible individual potter signatures.

The field experiment allowing to address these issues took place in the northern India region of Uttar Pradesh. In this region the pottery handicraft is a traditional activity, with the skill being transmitted vertically within endogamous castes that produce standardized traditional objects in mass production [10, 39]. Video-based data acquisition took place in the workshops of two potting communities working and living in the same village and mainly differentiated by the use of different wheels. The Prajapati potters used a hand-operated, high-inertia stick-wheel, while the Multani Kumhar potters used a foot-operated, low-inertia kick-wheel. Both communities used the same soft grey clay to produce a similar repertoire of traditional vessel types for a common market.

All seven participating potters were confirmed experts with more than ten years of experience on the task. Among the four potters from the Prajapati workshop (referred to as AR, BA, GA and KA), GA and KA were father and son; GA and AR were uncle and nephew. There was no close family relationship between the three potters from the Multani Kumhar workshop (KD, NA and YA). For the present purposes, each community was asked to select three preferred traditional pottery types, to be thrown by each potter in five specimens with self-selected quantities of clay. Prajapati potters selected the Money-bank, Handiya, and Kullar, while Multani Kumhar potters selected the Money-Bank, Handi, and Kulfi (see S1 Fig for graphical representations of these traditional vessel types). Therefore, in the following analyses we concentrated most of our attention on the Money-banks thrown by all participating potters. Corroborative evidence from the analyses of the four other vessels types is mainly presented as supporting information.

## Results

**Fig 1:**
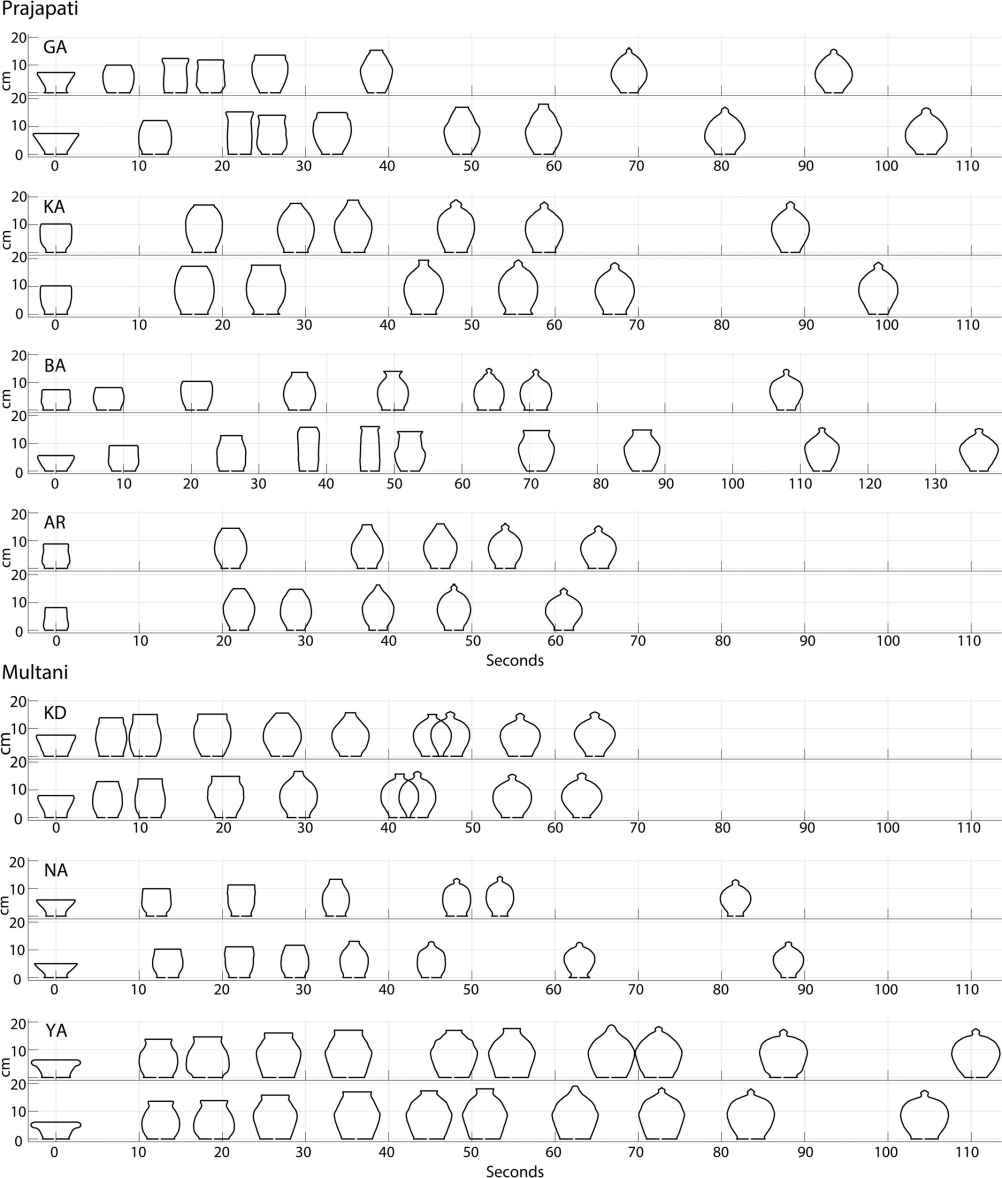
Morphological development of Money-bank vessels as a function of time. Two examples (trials 3 and 4) for Money-banks thrown by each of the Prajapati potters (top panel: GA, KA, BA, AR) and Multani potters (bottom panel: KD, NA, YA). Successive outlines on the timelines represent the vessel form after each fashioning gesture of the potter, from the initial pre-formed shape (t = 0) up to the final vessel shape. Size scale (height) is indicated on the y-axis. Note the slightly different time scale for BA.

In order to capture the morphological development towards the final form, for each vessel thrown we digitized its outline from video frames after every clay-deforming manual fashioning gesture. Fig 1 shows clay form as a function of time for two out of the five Money-bank trials performed by each of the seven potters (see S2 Fig for the full data set).

### Final vessel dimensions and size development

As allowed by our protocol based on self-selected quantities of clay, final vessel size varied over potters, with Multani potters YA and NA producing, respectively, the largest and the smallest Money-banks. ANOVAs on absolute final vessel dimensions (see Table 1) corroborated the observation that final Money-bank size was not community-specific, as all *F*’s (1,5) < 1 and effect sizes were small (η^2^_g_ = 0.024, 0.056 and 0.005, respectively, for height, maximal diameter and exterior surface area).

**Table 1:** Absolute dimensions of final vessels. Means and coefficients of variation (100*SD/M, between parentheses) of height (H), maximal diameter (MD) and exterior surface area (ESA) of the final forms across trials for the traditional vessels thrown by each potter.

Vessel type		Prajapati				Multani Kumhar		
		GA	KA	BA	AR	KD	NA	YA
Money-bank	H (cm)	16.3 (5.4)	18.4 (2.3)	14.5 (5.9)	15.2 (2.0)	15.9 (2.7)	12.9 (4.8)	17.7 (3.1)
	MD (cm)	14.3 (4.2)	13.9 (2.4)	12.3 (8.7)	12.8 (4.5)	14.2 (3.4)	11.1 (1.3)	17.4 (2.1)
	ESA (cm^2^)	543.3 (8.3)	596.9 (4.6)	422.8 (13.2)	466.9 (8.5)	500.6 (5.5)	330.9 (6.2)	745.8 (4.1)
	H (cm)	13.9 (7.0)	13.8 (2.0)	12.7 (2.7)	13.2 (2.5)			
Handiya	MD (cm)	15.3 (8.3)	16.6 (1.1)	14.9 (3.9)	14.8 (1.9)			
	ESA (cm^2^)	587.0 (14.7)	640.0 (3.2)	525.4 (7.4)	557.0 (5.1)			
	H (cm)	14.7 (5.2)	12.5 (3.5)	14.4 (3.4)	14.2 (2.7)			
Kullar	MD (cm)	12.0 (3.6)	11.4 (2.0)	12.7 (3.5)	12.1 (5.2)			
	ESA (cm^2^)	513.8 (7.8)	413.3 (5.9)	528.8 (7.8)	473.1 (8.1)			
	H (cm)					12.1 (3.7)	12.7 (2.3)	14.3 (4.0)
Handi	MD (cm)					17.4 (1.5)	17.8 (1.2)	20.5 (1.9)
	ESA (cm^2^)					580.0 (3.6)	640.5 (2.7)	842.5 (5.5)
	H (cm)					9.2 (3.4)	8.33 (4.1)	9.7 (3.6)
Kulfi	MD (cm)					12.5 (2.3)	12.3 (3.6)	14.3 (2.0)
	ESA (cm^2^)					346.3 (3.5)	320.6 (6.7)	445.1 (4.4)

Considerable differences between potters were also observed for the time taken to fashion a vessel of a given type and for the number of manual fashioning gestures deployed (visible in Fig 1 as the number of digitized outlines on the trial timelines). Throwing larger Money-banks did not, however, systematically require more time, as indicated by the non-significant Pearson correlation between throwing duration and final vessel exterior surface area (*r*_33_ = 0.07, *P* = 0.68, 95% *CI* = [-0.269, 0.394]). Throwing duration was in fact shortest for Prajapati potter AR and Multani potter KD (*M* ± *SD* respectively 61.2 ± 2.8 s and 62.9 ± 6.0 s) and longest for Prajapati potter BA (155.8 ± 37.9 s). Throwing larger Money-banks was positively related to the number of fashioning gestures deployed (*r*_33_ = 0.41, *P* = 0.012, 95% *CI* = [0.092, 0.656]), varying from 5 or 6 for Prajapati potter AR to 10 or 11 for Multani potter YA and 8 to 12 for Prajapati potter BA.

As anticipated, within-potter variation in final vessel size was smaller than between-potter variation; for example, for Money-bank exterior surface area *F*(6,28) = 64.21, *P* < 0.0001. This observation, corroborated by the small (on average 4.2%) within-potter coefficients of variation of the absolute vessel dimensions (Table 1), supports the general finding that the outcome of expert behaviour is highly reproducible over repeated trials [15, 25, 40].

**Fig 2:**
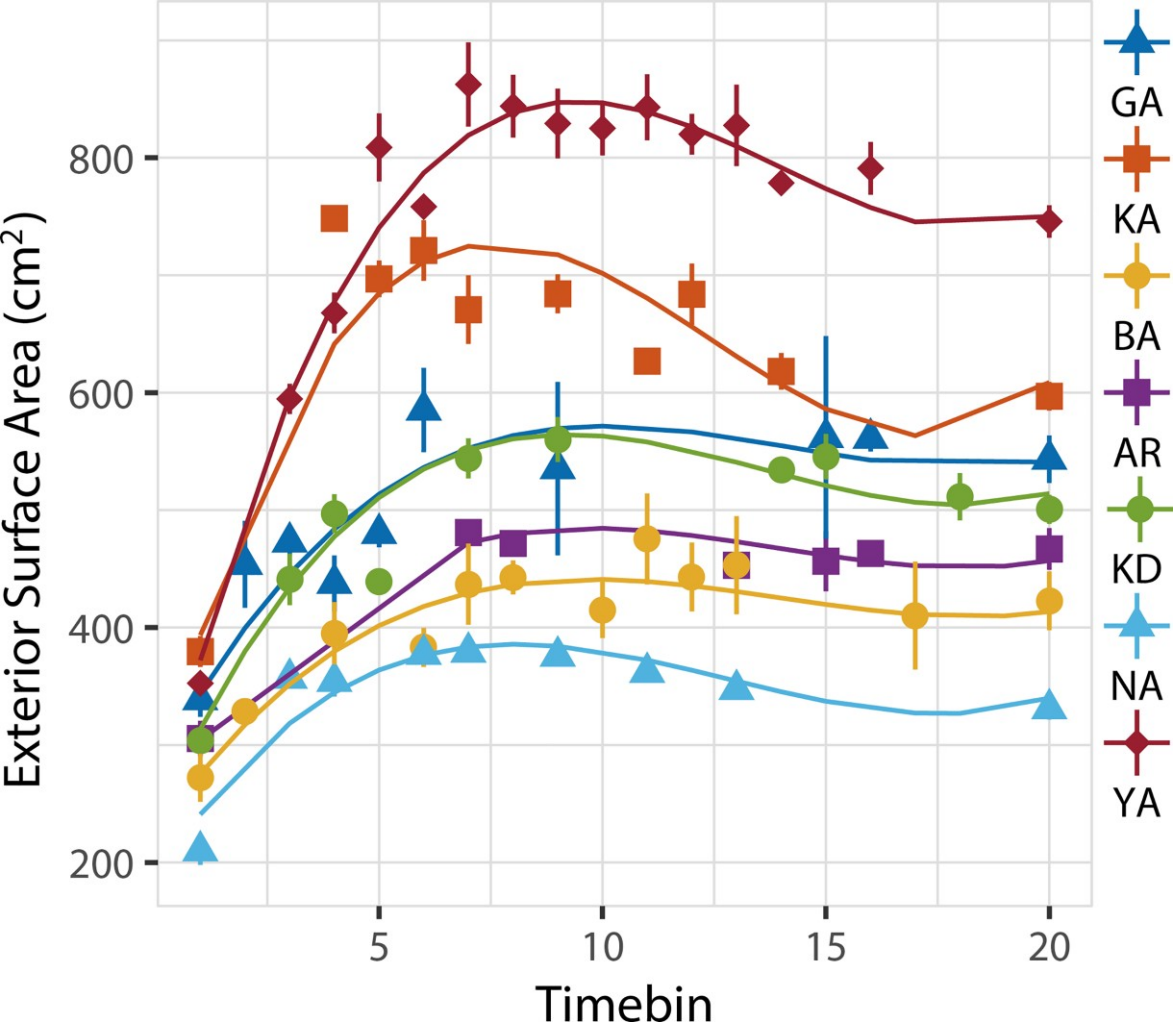
Growth curves of Money-bank exterior surface area. Symbols and curves indicate exterior surface area (ESA) for individual potters (Prajapati GA, KA, BA and AR; Multani Kumhar KD, NA and YA), represented as a function of 20-bin normalized time, from the pre-formed initial form (time bin 1) up to the final vessel form (time bin 20). Curves represent modeled vessel growth curves. Each symbol represents the mean ESA of the trials with one or more form points falling within a particular time bin. Error bars represent ±1 SE.

As can be seen from Fig 1, the Money-bank size development over time was generally characterized by a rapid initial increase, followed by a small decrease that gradually levelled out, sometimes reversing to a slight increase, towards the end of the throwing process. This pattern is clearly evident in the development of exterior surface area in the (20-bin) time-normalized representation of Fig 2 and could thus effectively be captured by an orthogonal 3rd-order polynomial growth curve model. Although inclusion of a random effect of individual-potter intercept significantly improved the model fit (likelihood ratio test: *χ*^2^(1) = 469.75, *P* < 0.0001), between-potter growth-curve differences could not be fully reduced to differences in initial size. Time trends in size space were potter specific, as demonstrated by a further improvement of the model (likelihood ratio test: *χ*^2^(9) = 144.21, *P* = 0.0001) by inclusion of a random effect for the slope of the three time-terms. We found no evidence for a community fixed effect (likelihood ratio test: *χ*^2^(4) = 7.91, *P* = 0.0949).

### Final vessel shape

Notwithstanding these differences in size, all potters produced clearly identifiable (i.e., type-specific) traditional forms (e.g., see last image on each row in Fig 1 for Money-banks). To quantify pure shape, we performed elliptical Fourier analysis on all digitized clay outlines and normalized the resulting Fourier coefficients to the first harmonic to correct for size [13, 37]. The full set of size-corrected Fourier coefficients was then subjected to a Principal Component (PC) analysis. For all evolving vessels over 80% of the total shape variance was captured by the first three PCs. Hence, the development of shape could adequately be traced out in this shared 3D shape-space.

**Fig 3:**
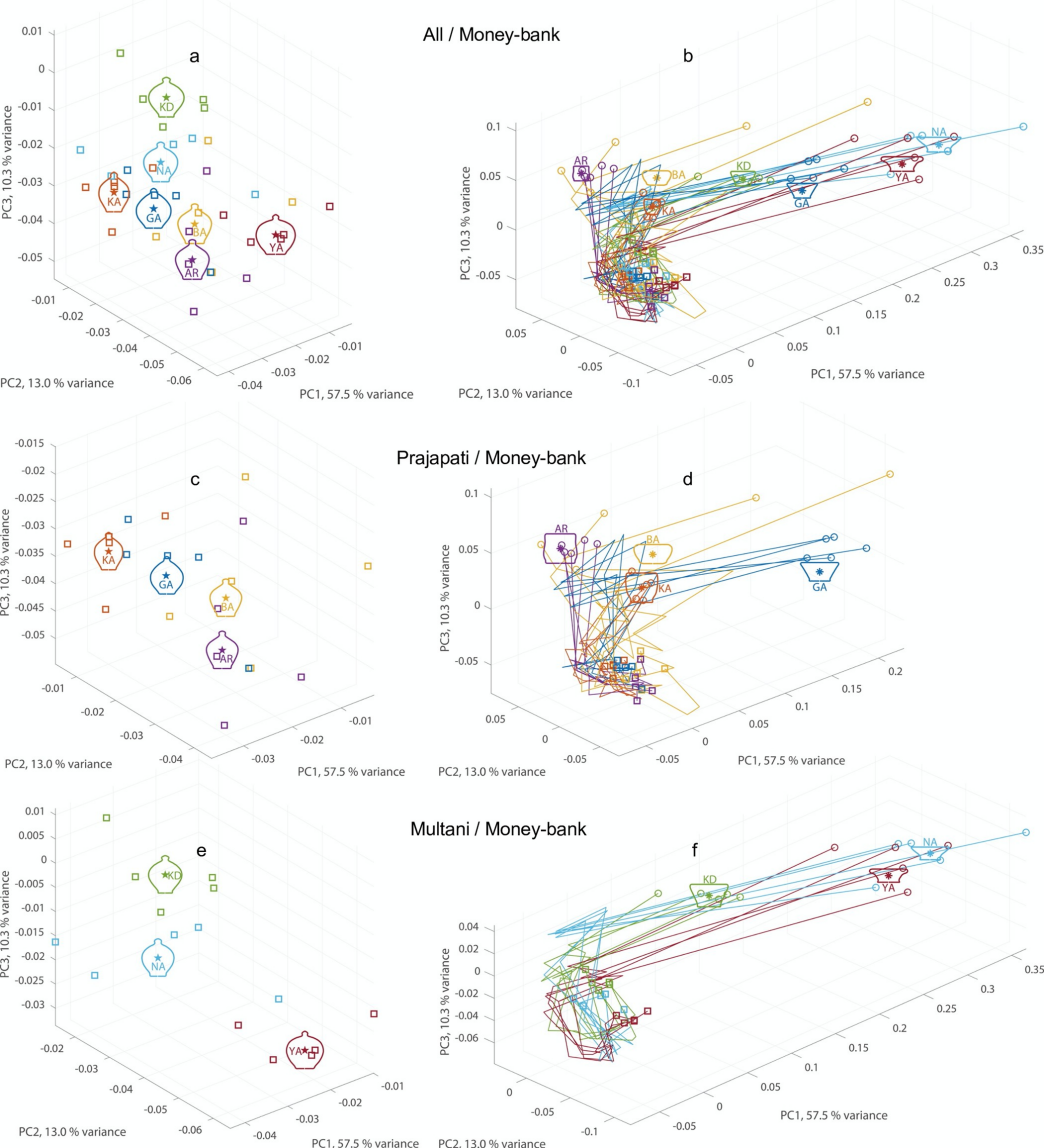
Development of Money-bank morphology in shape space. *Right panels*: Development of vessel morphology is represented as trajectories through 3D shape space, from the initial pre-formed shape (open circles) to the final shape (open squares), for the Money-bank vessels thrown by all seven potters (**b**), by Prajapati potters GA, KA, BA and AR (**d**) and by Multani Kumhar potters KD, NA and YA (**f**). Individual potters are colour-coded. For each potter mean initial shape is represented by an outline centered on the position indicated by an asterisk. *Left panels*: Zoom on final vessel shapes (open squares) revealing subtle between-potter differences. For each potter mean final shape is represented by an outline centered on the position indicated by an asterisk (**a**, **c** and **e**).

The five final Money-bank shapes thrown by each of the seven potters are presented in the left panels of Fig 3 (see S3 Fig for the other traditional vessel types). Despite the apparent overall similarity in shape across vessels produced by different potters, permutation tests revealed statistically significant heterogeneity among individuals for each traditional type (Table 2, “Final Shape” columns). These results demonstrated that different potters produced vessels with more morphometric variation among than within potters, both over communities and within communities. Corroborating the results of earlier work [37, 38], we therefore conclude that expert potters imprint subtle but identifiable individual (shape) signatures even on typical traditional types of vessel produced for a common consumer market.

**Table 2:** Results (*R*^2^ and *P*-values) of the permutation tests performed on the size-corrected coefficients resulting from elliptical Fourier analyses of the final and initial pre-formed clay shapes for each traditional vessel type thrown by the four Prajapati (PR) and the three Multani Kumhar (MK) potters. For each vessel type, within-potter effects are based on five trials, except for the Handiya where one trial was missing for PR potter AR.

Type	Component		Final Shape		Pre-formed Shape	
		*df*	*R*^*2*^	*P*	*R*^*2*^	*P*
Money-bank (PR & MK)	Potter	6	0.55	<0.0001	0.76	<0.0001
	Residuals	28	0.45		0.24	
Money-bank (PR)	Potter	3	0.41	<0.0001	0.65	<0.0001
	Residuals	16	0.59		0.35	
Money-bank (MK)	Potter	2	0.53	<0.0001	0.65	<0.0001
	Residuals	12	0.47		0.35	
Handiya (PR)	Potter	3	0.39	0.0007	0.58	0.0006
	Residuals	15	0.61		0.42	
Kullar (PR)	Potter	3	0.40	0.0005	0.24	0.1076
	Residuals	16	0.60		0.76	
Handi (MK)	Potter	2	0.38	0.001	0.61	<0.0001
	Residuals	12	0.62		0.39	
Kulfi (MK)	Potter	2	0.43	<0.0001	0.57	<0.0001
	Residuals	12	0.57		0.43	

### Vessel morphogenesis

Fig 1 shows that, except for potter BA, the Money-bank shaping processes (i.e., the morphological development) were fairly consistent over trials within individual potters (also see S2 Fig for the other vessel types). In contrast, we found large differences amongst potters in morphological development towards each final vessel form. In throwing the Money-banks, for example, after the second fashioning gesture Multani potter YA invariably produced a large shape with a rounded sphere-like bottom and a wide, rimmed aperture on the top. Then, the widest part of the vessel (i.e., where the diameter is maximal) moved progressively upward after each successive gesture, followed by the closing of the aperture on the top, resulting in a large, almost spherical final shape. In contrast, after the second fashioning gesture Prajapati potter GA produced almost cylindrical shapes with a rim around the aperture. The vessel’s profile subsequently became rounder with each successive gesture. Multani Potter NA produced less pronounced cylindrical shapes after a few gestures. Prajapati potter KA and Multani potter KD produced distinct though both barrel-like shapes after the first gestures. KA consistently produced elongated vessels, while KD produced wider vessels, with sharper curves at the maximal diameter height. Prajapati potter AR narrowed the aperture immediately after the second gesture, thereby rapidly approaching the final form.

**Fig 4:**
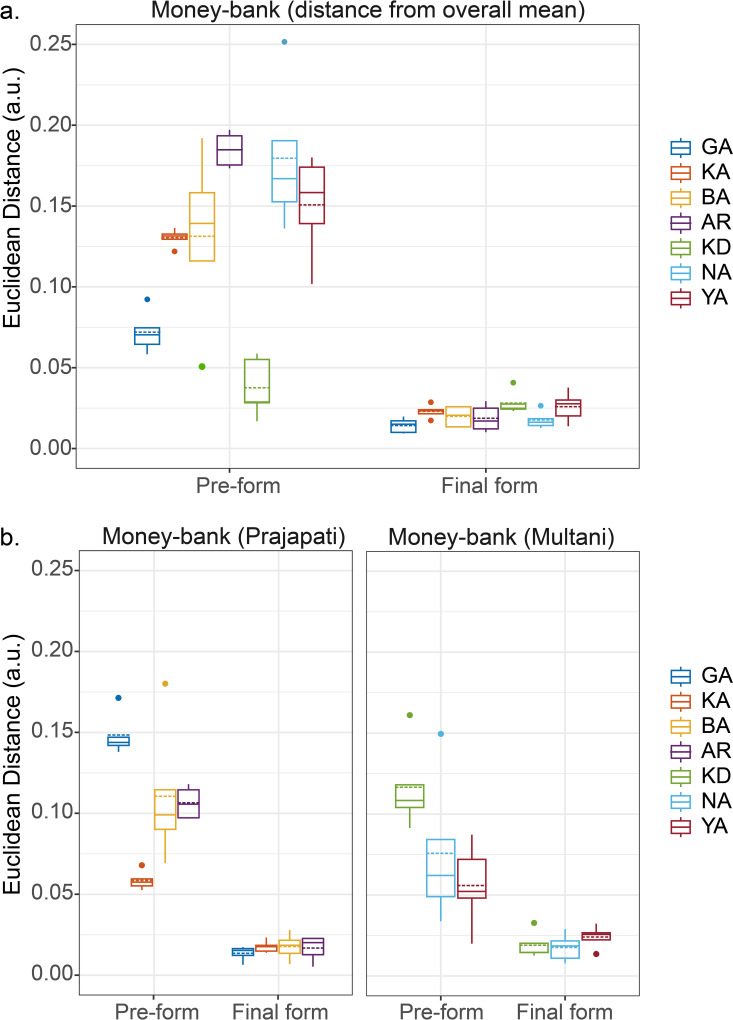
Euclidean distances (in shape space) from group mean Money-bank shapes. Boxplots represent individual-trial Euclidean distance from the group mean shape at the initial pre-formed stage and at the final stage for the Money-bank vessels thrown (**a**) by all seven potters and (**b**) by Prajapati potters GA, KA, BA and AR and Multani Kumhar potters KD, NA and YA separately. Note that at each stage the pertinent group mean shapes differ over the groups considered. Individual potters are colour coded. The solid and dotted lines in the box indicate the medians and means of the data, respectively.

Idiosyncrasies in morphological routes (towards the final shape) appeared not only at intermediary stages but were already present before the onset of the forming phase (t_0_ in Fig 1). As can be seen from the right panels in Fig 3 for the Money-banks, the starting positions of the trajectories (corresponding to the shape products of the pre-forming phase) varied markedly over potters; the same phenomenon was observed for the other traditional types (see S3 Fig). Permutation tests confirmed that, for each of the traditional types thrown except Kullar, among-potter variation in pre-formed clay shape was significantly larger than within-potter variation, both over communities and within communities (see Table 2, “Pre-formed Shape” columns), corroborating the idea that the expert potters have their own idiosyncratic ways of shaping the visibly standard, traditional vessels. Despite the significant differences among potters for both final shapes and pre-formed shapes, the individual-trial Euclidean distances from the group mean in the 3D shape-space computed at the initial and final stages indicated that potters started from considerably different locations in shape-space but all converged onto closely neighbouring shape-space locations at the final stage for Money-bank (see Fig 4) as well as for the other traditional vessel types (see S4 Fig). A linear mixed model ANOVA on the individual-trial Euclidean distances from the group mean in shape space with fixed-effects for the Stage factor and a random effect for the intercept for individual potter revealed significant effects of Stage for all vessel types (*P*’s < 0.001, see Table 3). These results provide unequivocal evidence that potters reliably followed distinctive individual routes through morphological space towards a much less variable final shape.

**Table 3:** Results (*F*, *df* and *P*-values) for the factor Stage of the linear mixed model analysis of the Euclidean distance from the grand mean in shape space, at the initial pre-formed stage and at the final stage, for each traditional vessel type thrown by the four Prajapati (PR) and the three Multani Kumhar (MK) potters. For each vessel type, analysis is based on five trials, except for the Handiya where one trial was missing for PR potter AR.

Type	*df*	*F (Stage)*	*P*
Money-bank (PR+MK)	62	1465.2	<0.0001
Money-bank (PR)	35	359.7	<0.0001
Moneybank (MK)	26	33.81	<0.0001
Handiya (PR)	33	161.5	<0.0001
Kullar (PR)	35	77.28	<0.0001
Handi (MK)	26	116.37	<0.0001
Kulfi (MK)	26	31.18	<0.0001

Taken together, the present results demonstrated that, in traditional wheel-throwing, the variation among potters in vessel morphogenesis (i.e., in the clay’s morphological transformation routes) is substantially greater than the subtle potter-specific variation remaining in the final shapes produced. This is true for all five traditional vessel types and both potter communities examined.

## Discussion

The shaping process as characterized by vessel morphogenesis data invalidates the high-fidelity copying hypothesis and is highly consistent with the deliberate practicing hypothesis. While we did not identify individual potter mentors (except for Prajapati GA’s son KA and nephew AR), we stress that within the community-of-practice perspective [22] a cultural model is taken to be shared by the community rather than being specific to each individual member. Yet, for each of the different vessel types studied participants followed clearly distinctive, individual routes through morphological space towards the much less variable final shape. Within specific communities, such variation amongst potters in vessel morphogenesis was observed whether close family relationships between participants existed (as in the Prajapati community) or not (as in the Multani Kumhar community). In addition, our results indicated that the number of fashioning gestures and the time required to fashion the vessels also significantly differed over participants. Thus, the seven participating potters demonstrated idiosyncratic fashioning styles that were equivalent for producing the same pottery types. Overall, these results provide empirical evidence in support of motor behaviour theory’s functional equivalence principle in the domain of pottery handicraft.

By focusing on the observable outcome of the elementary clay-deforming gestures our results reveal that cultural transmission of handicrafts does not entail near-perfect replication of elders’ way of doing although it does replicate the intended shape consistently and very well. We suggest that, at least at the level of elementary gestures [41–43], the proposition that fidelity-copying would underlie the emergence of skilled crafting behaviour is in fact fallacious. Learning a complex motor skill requires active exploration of the constraints and opportunities offered by the environment for achieving the task at hand. To truly appreciate craftmanship it is therefore essential to realize that the difficulty of the task implies that it requires such individual skill learning to recur across generations [43]. This is not, of course, to say that cultural transmission plays no role in handicrafts. As highly specialized motor behaviours, handicrafts could hardly be acquired on the basis of individual learning alone [1]. The learning environment itself is already clearly socially structured, with the presence of typical tools and materials, finished vessels, active potters, etcetera. Social learning and transmission are thus no doubt key mechanisms operating to retain craft traits over generations. What we suggest is that the conceptualisation of cultural transmission by way of fidelity-copying poorly grasps these intricate mechanisms of skill learning [44].

Students of sociocultural learning have pointed out that when newcomers (whether they be children or apprentices) learn a craft or other cultural motor skill, the interaction with elders mastering the task guides participation in the practice and scaffolds the learning process [3, 22, 45, 46]. In so doing, the cultural environment provides social pointers that orient and channel the learner’s attention, allowing perceptuomotor exploration to occur over an optimal area of the task space [47–50]. This social channelling helps the learner to perceive the relevant task space properties and to progressively exploit them functionally through practice. The functional (i.e., effective) aspects of motor skill thereby acquired correspond to the bodily actions that cause the intended effects on the environment. Importantly, these functional aspects generally do not correspond to observable body configurations and kinematics but rather to the fine tunings of interaction kinetics. In the case of pottery throwing the functional aspects correspond to the tuning of the pressure forces exerted by the hands so as to plastically deform particular areas of the clay body; in the case of stone knapping [42] or flaking [43] they correspond to the tuning of the hammer’s kinetic energy transferred onto particular locations of the handheld platform so as to detach a flake. What matters here is to acknowledge that the functional aspects of motor skill mastered by experts are not observable, nor truly verbalizable; they correspond to know-how, non-discursive embodied knowledge [51] that cannot be transmitted or copied independent of its application in the world [52]. The functional aspects of the task need to be discovered through individual, hands-on exploration [30].

What may be reproduced during social learning are the formal (i.e., non-effective) aspects of the skill. Contrary to functional aspects, formal aspects and their sequencing are observable and can be verbalized, captured in action recipes and characterized by words or images. At the level of the handicraft’s elementary gestures, such formal aspects correspond to body segment positions and kinematics. Importantly, these aspects do not determine the result of the skill as the functional aspects do. In the case of wheel-throwing the formal aspects related to the elementary gestures notably include the hand positions successively used in fashioning a vessel. These hands positions do not determine the manual pressure forces exerted and thereby the vessel shape [13]. Yet, as revealed by personal ethnographic observations in different potting communities, mentors typically encourage their apprentices to carefully watch the hands positions used so that they can reproduce them. The existence of culturally specific hand positions in French, Nepalese and Indian professional potting groups [14] attests that potters do reproduce the hands positions they have observed during their learning. Still, the co-existence of such culturally specific hand positions with both idiosyncratic and cross-cultural hand positions indicates that potters do not limit their repertoire to the cultural hand positions observed in elders.

As a cautionary remark, we emphasize that formal copying should not be conflated with the true learning of the skill. Without active engagement in deliberate practice to explore the task space, strict formal copying will not lead to the emergence of skill. However, formal copying of observable aspects of a mentor’s behaviour may be expected to serve as a social pointer usefully channelling the learner’s activity. Formal copying can thus be understood as a method of facilitating the apprenticeship but not as a direct route to mastering the task constraints. Even when learning is facilitated by formal copying of a model, the necessary active exploration of the functional aspects of the task space will give rise to individual differences in skill ultimately acquired. As acknowledged by Forte [53], performance on a cultural motor skill may thus be understood as “a growing interaction between the transmission of knowledge and the development of manual practice” (p. 1).

We conclude that, notwithstanding its apparent explanatory power, fidelity-copying does not form the principal basis of cultural transmission of handicraft and other activities rooted in complex motor skills. The finding of highly similar artefacts should not be taken as evidence for a highly standardized transformation process, since fidelity-copying is not the fundamental mechanism by which cultural motor skills are transmitted; the present results instead fit much better with the alternative scenario of culturally specific skills that takes into account the complexity of skill learning, in which individuals are given opportunities for discovering the functional requirements of the task and building up their own ways of coping with them. Continuity in behavioural traditions is in fact possible because each new generation of experts learns to control the end-result of the task (i.e., what they want to produce). Expert potters can accurately reproduce different vessel shapes, whether they are acquainted with them or not [54]. This ability to control the characteristics of the final vessel thrown (including not only form but also resistance to collapse [55]) allows for the perpetuation and long-term development of potting traditions under the influence of a myriad of socio-economical and psychological factors, including production habits, market demand and social conformism among craftsmen of a given community of practice.

By highlighting the necessity of extensive individual deliberate practice in the acquisition of technical motor skills, the present study underscores that variations detected in the material record should not be considered as uniquely resulting from perception and/or dexterity-related error in copying a model [17]; material variation in artifacts also results from inter-individual differences in solutions for equivalent motor problems, discovered during apprenticeship. Another implication of our findings is that the relation between assemblage standardization and homogeneity of underlying manufacturing patterns is far from univocal. One should bear in mind that vessels falling into homogeneous geometrical types are not produced by culturally standardized fashioning patterns but by behaviours containing both cultural and individual features. The pool of individual behavioural variants existing in a community of practice is may constitute a source of change in artefacts.

## Methods

### Participants

Following visits during which the project was presented, participants were recruited within two traditional pottery workshops (one Prajapati and one Multani Kumhar) located in the same village in the Bulandshar district of the northern India state of Uttar Pradesh. In both communities, the throwing of the pots is traditionally performed by men only. After being duly informed, individual potters voluntarily decided to take part in the study. They were financially compensated for their participation. The participants were right-handed men over 25 years of age (*Mean* ± *SD*, Prajapati: 41.3 ± 14.9 yrs and Multani Kumhar: 33.7 ± 4.5 yrs) and had a minimum of ten years of wheel-throwing experience (Prajapati: 24.3 ± 14.5 yrs; Multani Kumhar: 21.0 ± 6.1 yrs). Potters from both participating workshops regularly produced at least five different kinds of everyday objects for the same local market, often in different sizes.

### Ethics statement

The study consisted in non-invasive behavioural observations of potters in their habitual workshops. Potters gave informed written consent prior to participation and were paid for their participation according to the local rates of the profession. These observations were made in the framework of E.G.’s Ph.D. project at Aix-Marseille University (France). According to operative French law (1988 Huriet-Serusclat law, amended in 2004) on the protection of persons in biomedical research, such a protocol did not require the approval of an ethics committee. The study was carried out in accordance with the ethical standards of the Declaration of Helsinki, the University of Aix-Marseille (E.G.’s affiliation at the time) and the Indian Anthropological Association.

### Procedure

Potters from each workshop were asked to select three preferred traditional pottery types, to be thrown by each potter in their usual conditions of practice in five specimens with self-selected quantities of clay. Prajapati potters selected the Money-bank, Handiya, and Kullar, while Multani Kumhar potters selected the Money-Bank, Handi, and Kulfi (see S1 Fig for graphical representations of the vessel types).

The experimental sessions were video-recorded under standardized conditions using a Panasonic NV-GS320 camcorder. The camera was fixed on a tripod with lens orientation centred on the vertical rotation axis of the wheel. The camera was positioned at a height of 30 cm above the level of the wheel at a horizontal distance of 4–6 m. The lower edge of the video scene was aligned with the centre of the wheel. The zoom was adapted to fully cover a 36-cm high by 42-cm wide calibration object (inverted T-shape) placed on the wheel at the start of each recording.

Of the total of 105 vessels thrown (seven potters, each throwing five specimens of three different vessel types), one (a Handiya vessel thrown by Prajapati potter AR) could not be analysed due to problems with the video-recording.

### Data analysis

For each trial, the images of the clay body profile after each fashioning gesture were extracted from the video frames (image resolution: 720 x 576 pixels; video sampling frequency: 25 fps). The first image captured the profile immediately following the (centring and opening) pre-forming phase and the last image captured the final profile; the intervening images captured the intermediate profiles during form development. This succession of profiles captured the vessel’s morphogenesis. The overall duration of the forming process and the total number of fashioning gestures per trial were also analysed as global variables describing the potters’ performances.

From the images we extracted the 2D coordinates of the right-half of the cross-sectional profiles by tracing them out on a Cintiq 21UX Wacom^®^ (Kazo, Japan) tablet with integrated screen. All further analyses were performed using Matlab^®^ (MathWorks, Natick MA, USA). The profile coordinates were converted from pixels to centimetres using a calibration factor obtained from the digitized dimensions of the calibration object. The profiles were re-sampled to generate an equal number of (256) points at regular height intervals along the vertical (Y) axis and the resulting coordinates were smoothed with a low-pass filter [13, 54]. Because wheel-thrown vessels are typically axisymmetric, profiles were subsequently converted to full pot outlines by multiplying the horizontal (X) coordinates by -1 to create the corresponding left edge.

Vessel dimensions were quantified by height, maximum diameter and exterior surface area. The latter was used as an overall measure of size.

To capture the development of size, the five trials of each potter throwing the Money-bank were time-normalized by dividing the duration between the first and the last fashioning gesture of each trial into 20 equally spaced time bins, and by representing the time of each stage of morphogenesis as *i*th bin within which the observed shape occurred. Growth curve models with third-order orthogonal polynomials using the maximum likelihood estimates [56] were fitted to the development of exterior surface area.

In order to quantify vessel shape, each outline was subjected to an elliptical Fourier transformation [13, 37, 57]. The resulting series of pairs of coefficients were normalized with respect to the first coefficients to correct for size differences [57, 58]. For graphical representation of the shape the size-corrected Fourier coefficients were subjected to a Principal Component (PC) analysis. Since over 80% of the full-dataset (857 outlines) total variance was captured by the first three components (57.5%, 13.0% and 10.3%, respectively), each particular shape could thus be represented as a point in a unique 3D PC space, allowing qualitative (visual inspection) and quantitative (numerical) comparisons of shape similarities and differences.

### Statistical analysis

For all statistical tests the (two-sided) alpha level used was 0.05. Differences between communities (Prajapati and Multani Kumhar) in height, maximum diameter and external surface area of the Money-bank vessels thrown were examined using Community x Trial ANOVAs with repeated measures on the last factor. Effect size of the Community factor was determined using generalized eta-squared (η^2^_g_) [59]. Relationships between the size of the vessel thrown (using the external surface area measure), on the one hand, and throwing duration and number of fashioning gestures deployed, on the other hand, were assessed using Pearson correlations and the associated 95% confidence intervals. In the growth model analysis of vessel size, the effects of adding the individual-potter random effects for the intercept and the slope, and the fixed effect of community on the improvement in model fit were tested using the likelihood ratio test which treats the change in a deviance measure (-2 log likelihood) as a chi-square statistic [60]. Statistical differences among shapes were examined using permutation tests [61] on the normalized Fourier Coefficients scores for each of the five shape classes separately using the adonis function in the R package vegan [62]. This analysis, performed at the initial pre-formed and final stages of shape development, tested for heterogeneity of shapes among the potters within shape types; if significant this test indicates the presence of individual influences on shape. Using the lme function in the R package nlme [63], the proximity of locations in the 3D shape-space (as measured by the individual-trial Euclidean distances from the mean location in shape space) were modelled using a linear mixed model with a fixed-effect for the Stage factor (initial pre-formed stage vs. final stage) and a random intercept effect for individual potter. A variance function (varIdent of nlme package) that allows different variances per stratum for each individual potter was used to model heteroscedasticity. The analysis was conducted for each of the five vessel types separately. For the Money-bank, the individual-trial Euclidean distance from the 7-potter overall mean as well as that from the mean locations of the Prajapati and Multani Kumhar communities were analysed separately.

## Supporting information

S1 Fig Customary traditional vessel types thrown. (PDF)

S2 Fig Morphological development of all vessels thrown by each individual potter. 7 pages., 1 potter per page.
(PDF)

S3 Fig Development of vessel morphology in shape space for other traditional vessel types. (PDF)

S4 Fig Euclidean distances (in shape space) from group mean shapes for other traditional vessel types. (PDF)
