# Supplementary material for: Traditional craftspeople are not copycats: Potter idiosyncrasies in vessel morphogenesis
Source: PLoS One. 2020 Sep 22;15(9):e0239362. doi: 10.1371/journal.pone.0239362 (PMC7508384; doi:10.1371/journal.pone.0239362)

S1 Figure. Customary traditional vessel types thrown, reconstructed for each type from the shape data as means over all potters concerned.
Prajapati potters threw five specimens of the Money-bank (**a**), Handiya (**b**) and Kullar (**c**). Multani Kumhar potter threw five specimens of the Money-bank (**a**), Handi(**d**) and Kulfi (**e**).

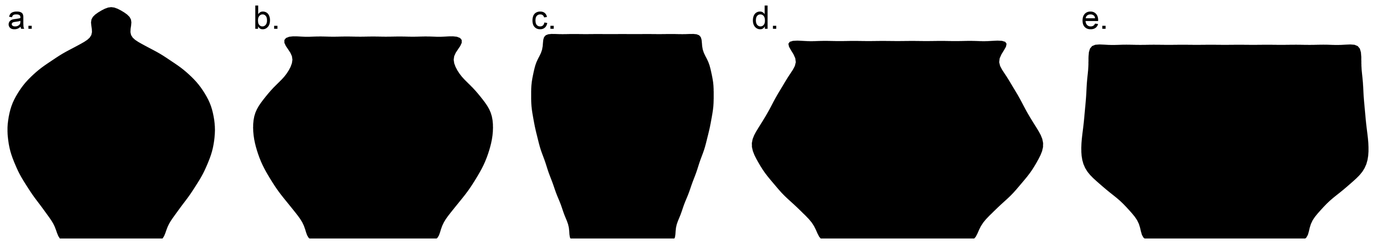

Supplement: S1 Fig — (PDF) [file pone.0239362.s001.pdf]
