# Supplementary material for: Traditional craftspeople are not copycats: Potter idiosyncrasies in vessel morphogenesis
Source: PLoS One. 2020 Sep 22;15(9):e0239362. doi: 10.1371/journal.pone.0239362 (PMC7508384; doi:10.1371/journal.pone.0239362)

S2 Figure (7 p., 1 potter per page): Vessel form development as a function of time for all vessels thrown by Prajapati potters GA (**a**), KA (**b**), BA (**c**), AR (**d**) and Multani potters KD (**e**), NA (**f**) and YA (**g**). Successive outlines on the time lines represent the vessel form after each fashioning gesture of the potter, from the pre-formed initial shape (t = 0) up to the final vessel shape. Size scale (height) is indicated on the y-axis. Note the different time scale for BA (**c**).

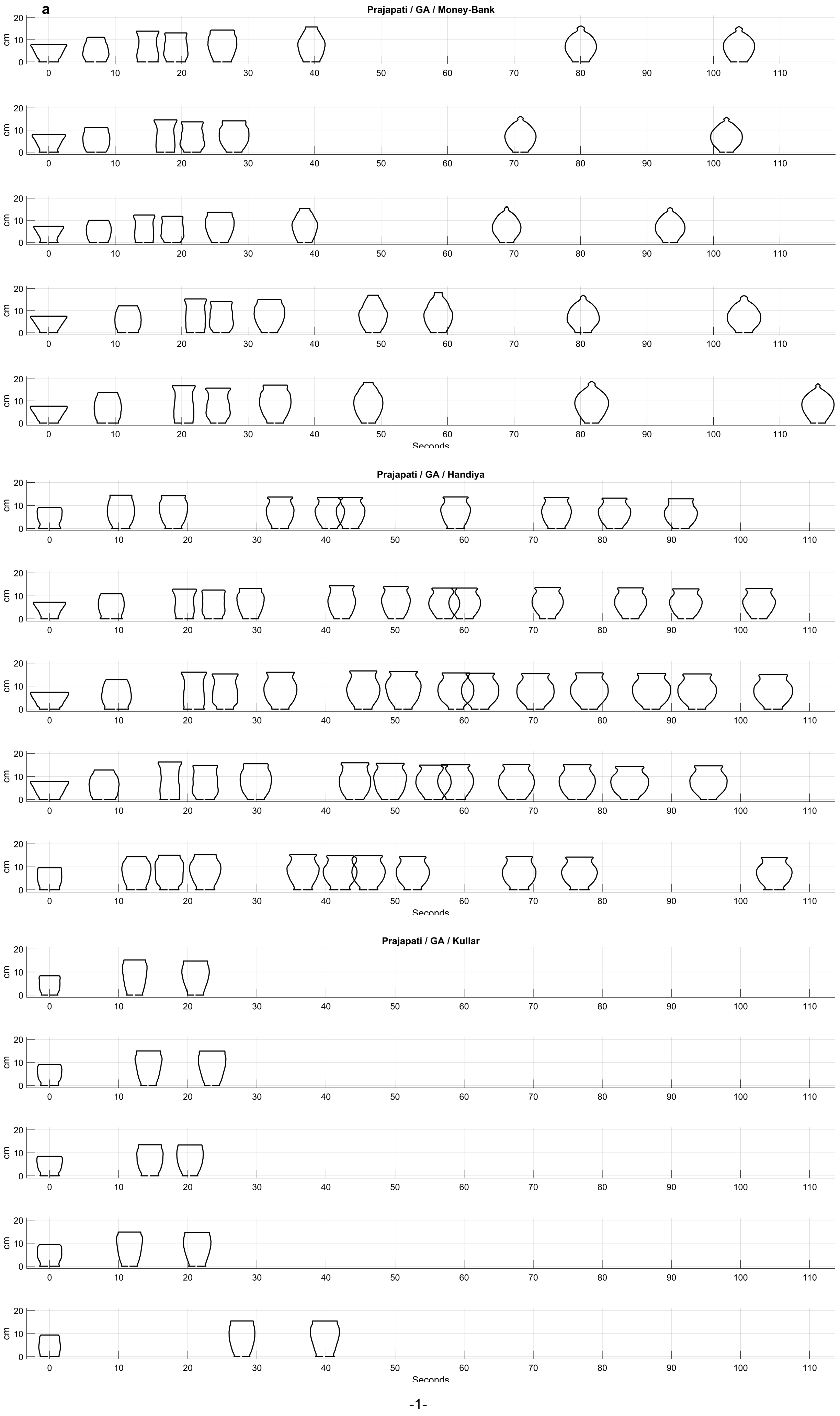

S2 Figure (7 p., 1 potter per page): Vessel form development as a function of time for all vessels thrown by Prajapati potters GA (**a**), KA (**b**), BA (**c**), AR (**d**) and Multani potters KD (**e**), NA (**f**) and YA (**g**). Successive outlines on the time lines represent the vessel form after each fashioning gesture of the potter, from the pre-formed initial shape (t = 0) up to the final vessel shape. Size scale (height) is indicated on the y-axis. Note the different time scale for BA (**c**).

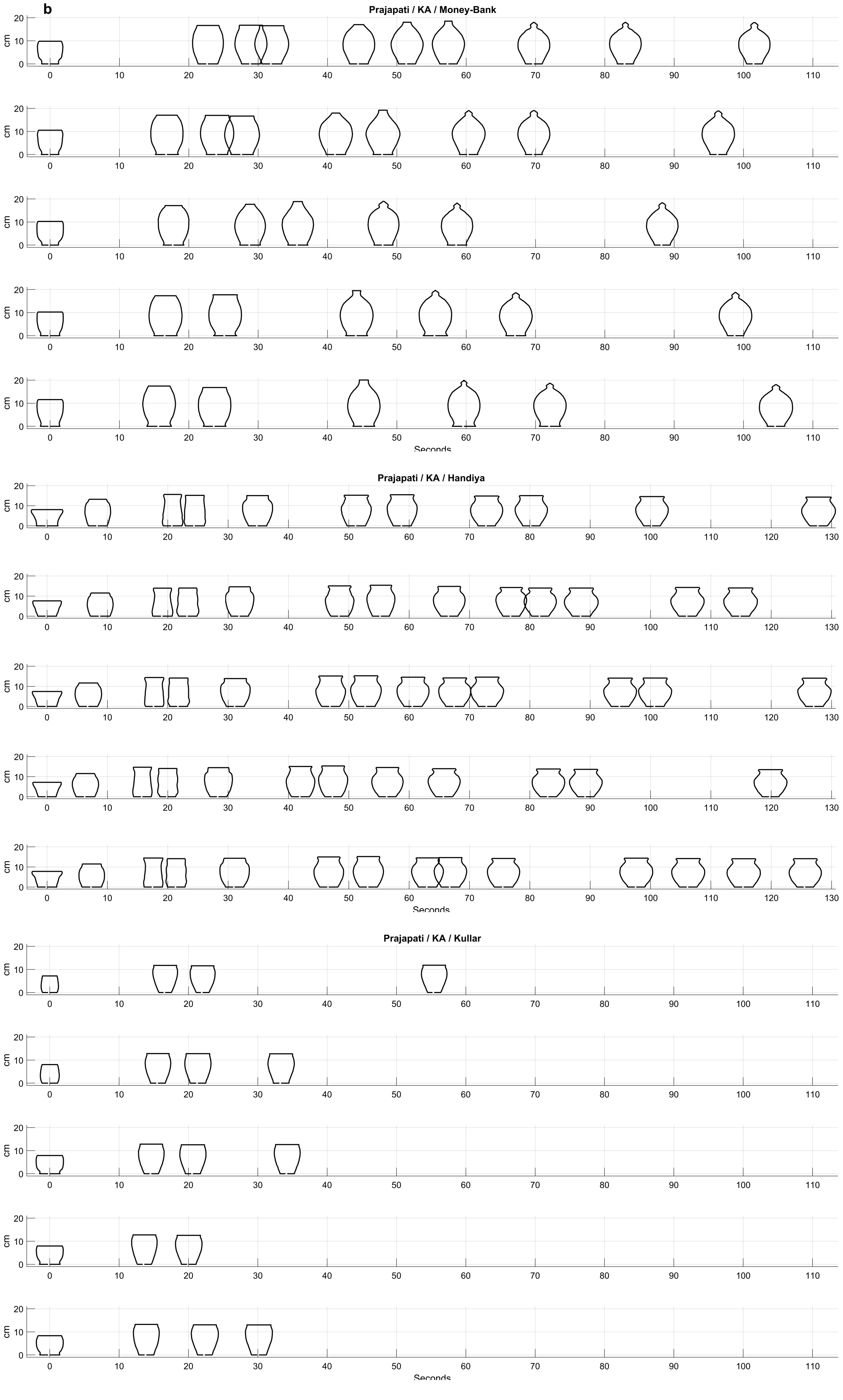

S2 Figure (7 p., 1 potter per page): Vessel form development as a function of time for all vessels thrown by Prajapati potters GA (**a**), KA (**b**), BA (**c**), AR (**d**) and Multani potters KD (**e**), NA (**f**) and YA (**g**). Successive outlines on the time lines represent the vessel form after each fashioning gesture of the potter, from the pre-formed initial shape (t = 0) up to the final vessel shape. Size scale (height) is indicated on the y-axis. Note the different time scale for BA (**c**).

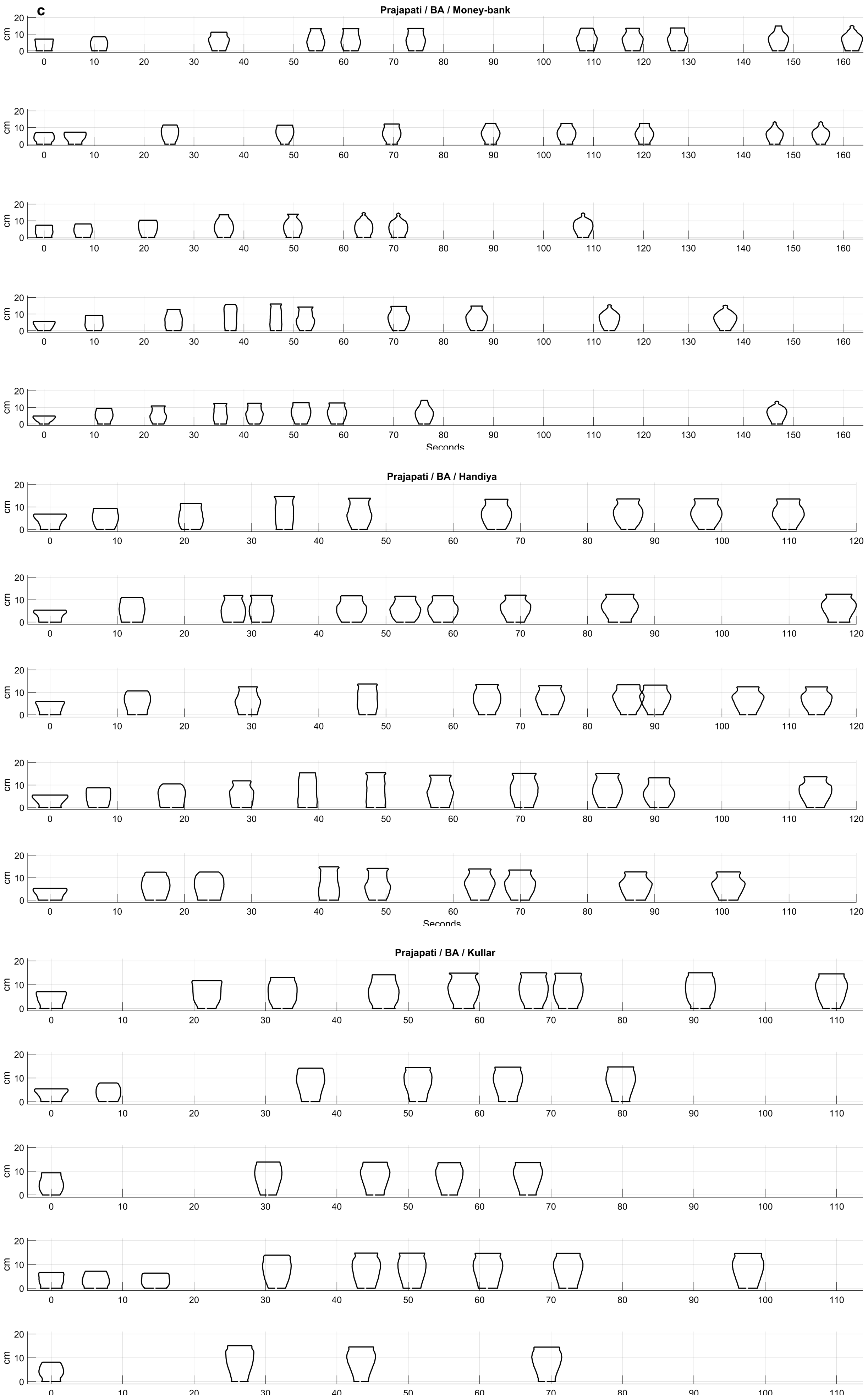

S2 Figure (7 p., 1 potter per page): Vessel form development as a function of time for all vessels thrown by Prajapati potters GA (**a**), KA (**b**), BA (**c**), AR (**d**) and Multani potters KD (**e**), NA (**f**) and YA (**g**). Successive outlines on the time lines represent the vessel form after each fashioning gesture of the potter, from the pre-formed initial shape (t = 0) up to the final vessel shape. Size scale (height) is indicated on the y-axis. Note the different time scale for BA (**c**).

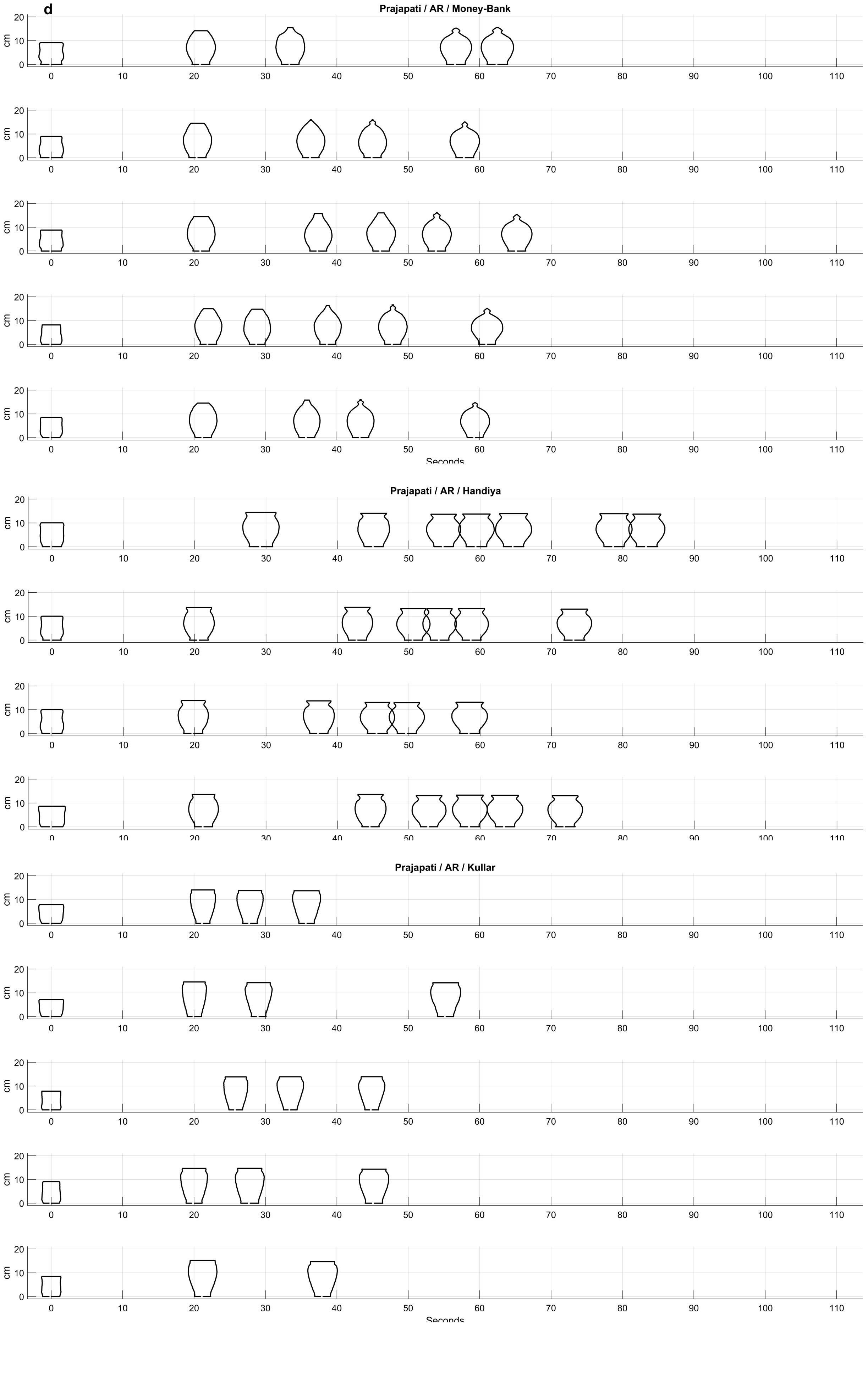

S2 Figure (7 p., 1 potter per page): Vessel form development as a function of time for all vessels thrown by Prajapati potters GA (**a**), KA (**b**), BA (**c**), AR (**d**) and Multani potters KD (**e**), NA (**f**) and YA (**g**). Successive outlines on the time lines represent the vessel form after each fashioning gesture of the potter, from the pre-formed initial shape (t = 0) up to the final vessel shape. Size scale (height) is indicated on the y-axis. Note the different time scale for BA (**c**).

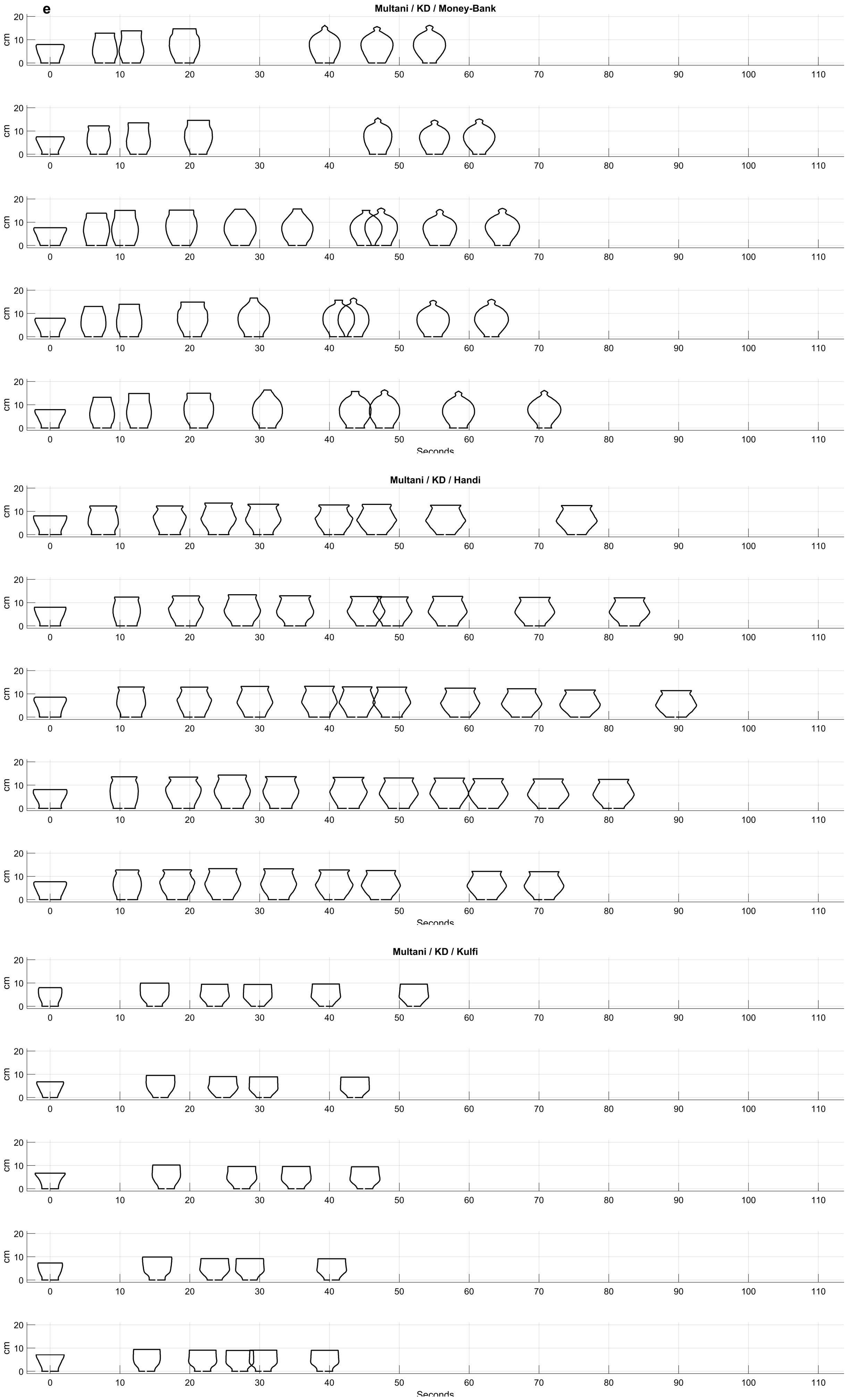

S2 Figure (7 p., 1 potter per page): Vessel form development as a function of time for all vessels thrown by Prajapati potters GA (**a**), KA (**b**), BA (**c**), AR (**d**) and Multani potters KD (**e**), NA (**f**) and YA (**g**). Successive outlines on the time lines represent the vessel form after each fashioning gesture of the potter, from the pre-formed initial shape (t = 0) up to the final vessel shape. Size scale (height) is indicated on the y-axis. Note the different time scale for BA (**c**).

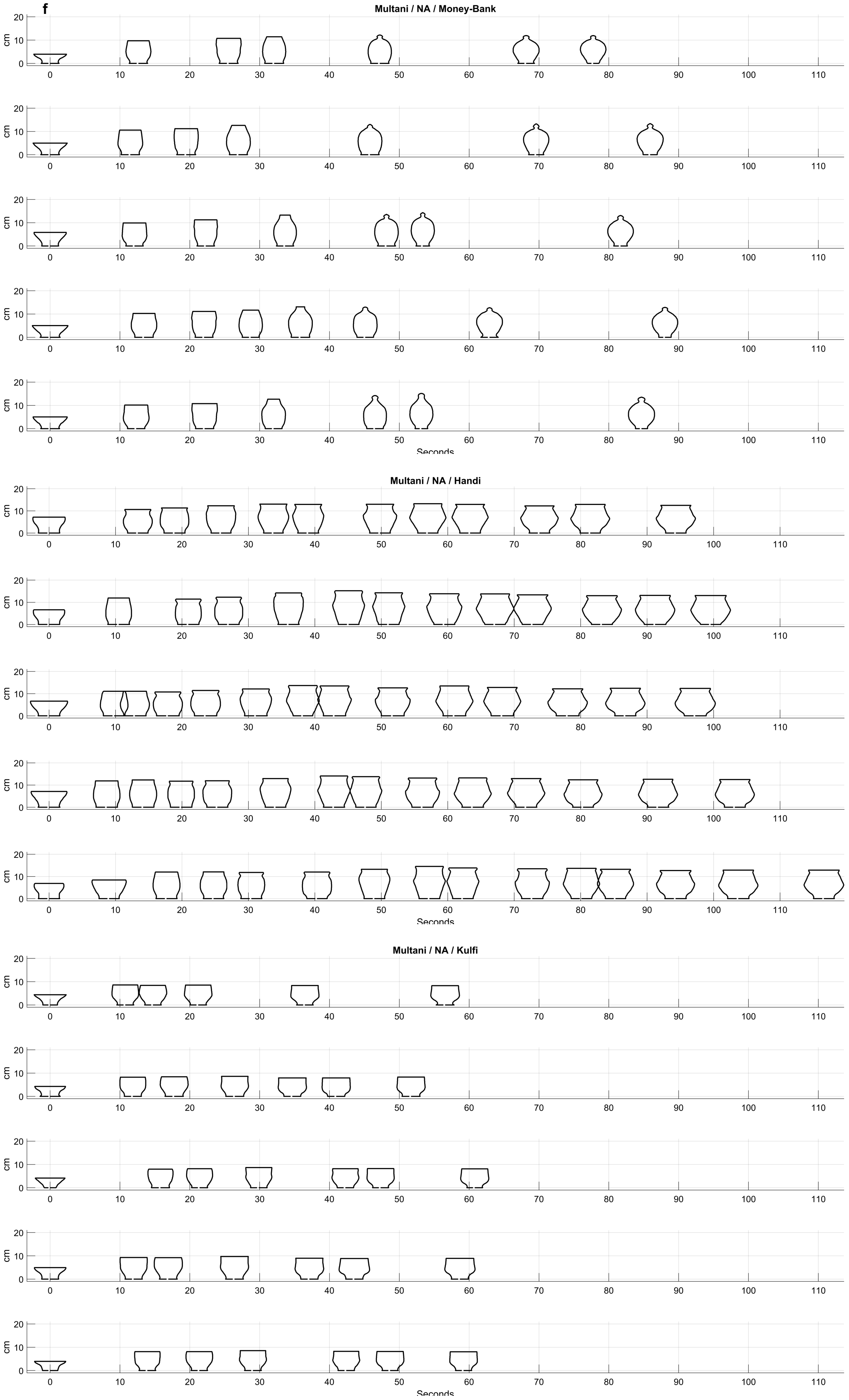

S2 Figure (7 p., 1 potter per page): Vessel form development as a function of time for all vessels thrown by Prajapati potters GA (**a**), KA (**b**), BA (**c**), AR (**d**) and Multani potters KD (**e**), NA (**f**) and YA (**g**). Successive outlines on the time lines represent the vessel form after each fashioning gesture of the potter, from the pre-formed initial shape (t = 0) up to the final vessel shape. Size scale (height) is indicated on the y-axis. Note the different time scale for BA (**c**).

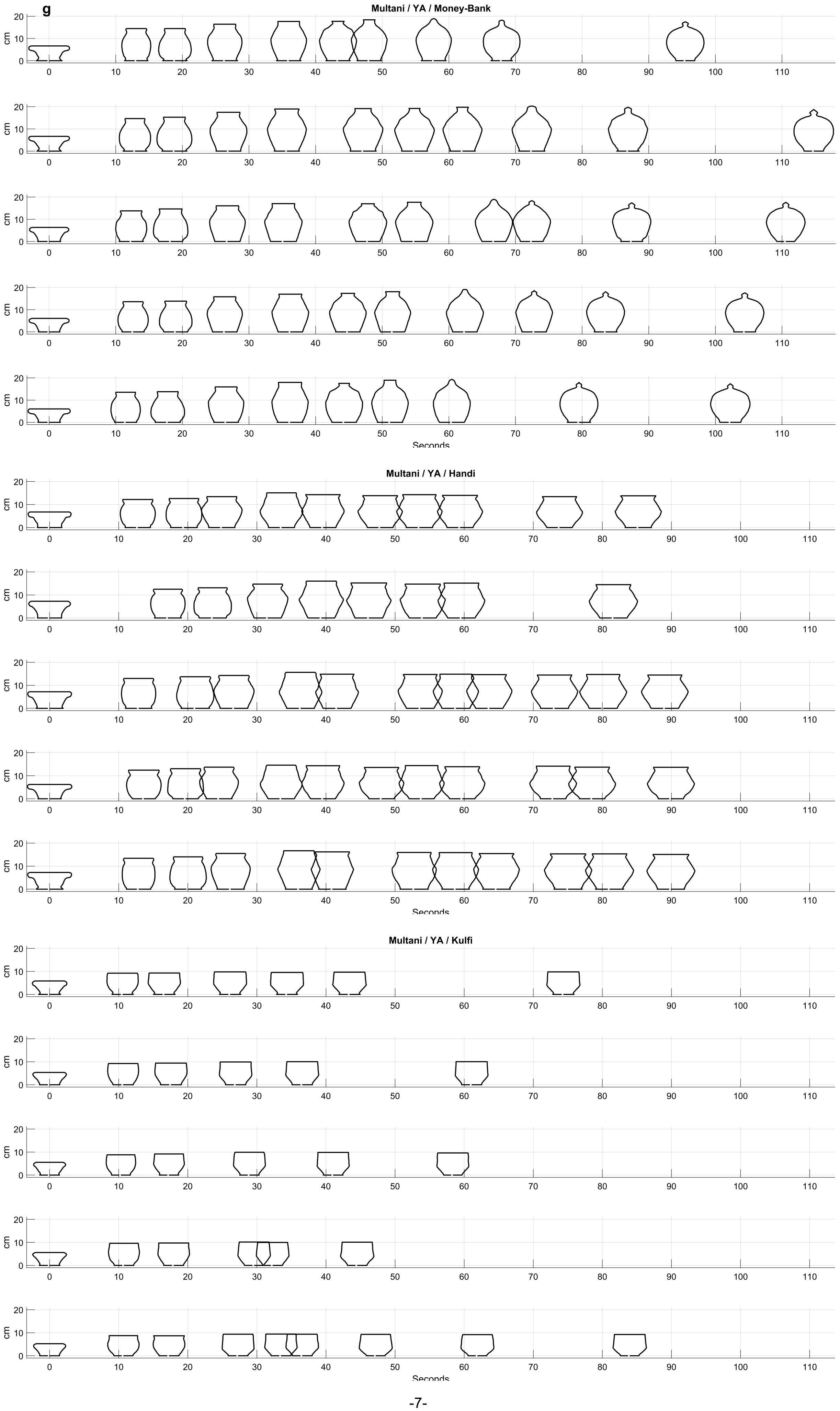

Supplement: S2 Fig — 7 pages., 1 potter per page. (PDF) [file pone.0239362.s002.pdf]
