# Supplementary material for: Traditional craftspeople are not copycats: Potter idiosyncrasies in vessel morphogenesis
Source: PLoS One. 2020 Sep 22;15(9):e0239362. doi: 10.1371/journal.pone.0239362 (PMC7508384; doi:10.1371/journal.pone.0239362)

S3 Figure. Development of vessel morphology in shape space for other traditional vessel types. *Right panels:* Development of vessel morphology represented as trajectories through 3D shape space, from the pre-formed initial shape (open circles) to the final shape (open squares), for the Handiya (b) and Kullar (d) vessels thrown by Prajapati potters GA, KA, BA and AR and for the Handi (f) and Kulfi (h) vessels thrown by Multani Kumhar potters KD, NA and YA. Individual potters are colour-coded. For each potter mean initial shape is indicated by an asterisk and outline. *Left panels:* Zoom on final vessel shapes revealing subtle between-potter differences (a, c, e, g). For each potter mean final shape is represented by an outline centred on the position indicated by an asterisk.

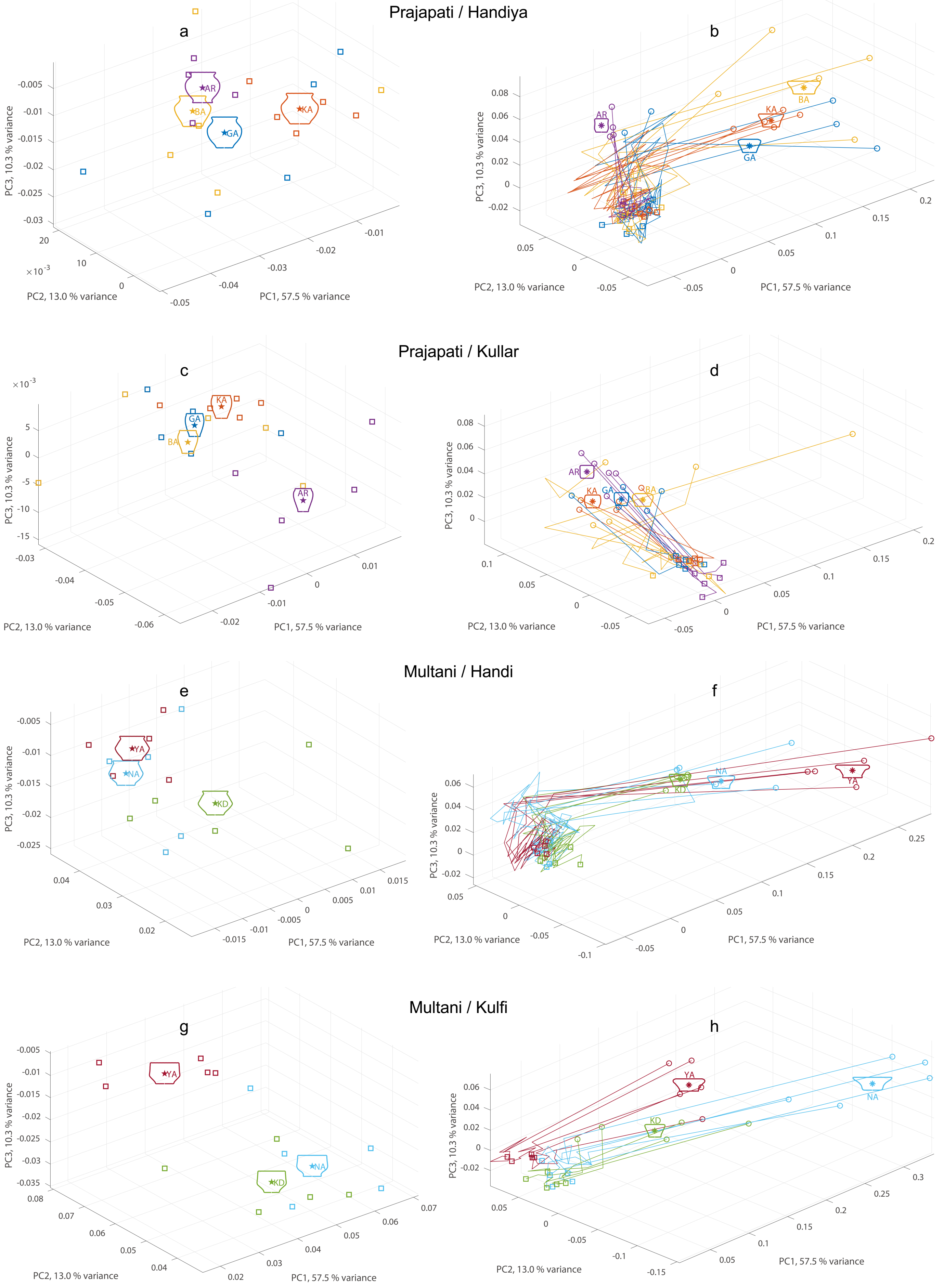

Supplement: S3 Fig — (PDF) [file pone.0239362.s003.pdf]
