# Supplementary material for: Traditional craftspeople are not copycats: Potter idiosyncrasies in vessel morphogenesis
Source: PLoS One. 2020 Sep 22;15(9):e0239362. doi: 10.1371/journal.pone.0239362 (PMC7508384; doi:10.1371/journal.pone.0239362)

S4 Figure. Euclidean distances (in shape space) from group mean shapes for other traditional vessel types. Boxplots of individual-trial Euclidean distance from the group mean shape at the initial pre-formed stage and at the final stage for (**a**) the Handyia and Kullar vessels thrown by Prajapati potters GA, KA, BA and AR and (**b**) for the Handi and Kulfi vessels thrown by Multani Kumhar potters KD, NA and YA. Individual potters are color-coded. The solid and dotted lines in the box indicate the medians and means of the data, respectively.

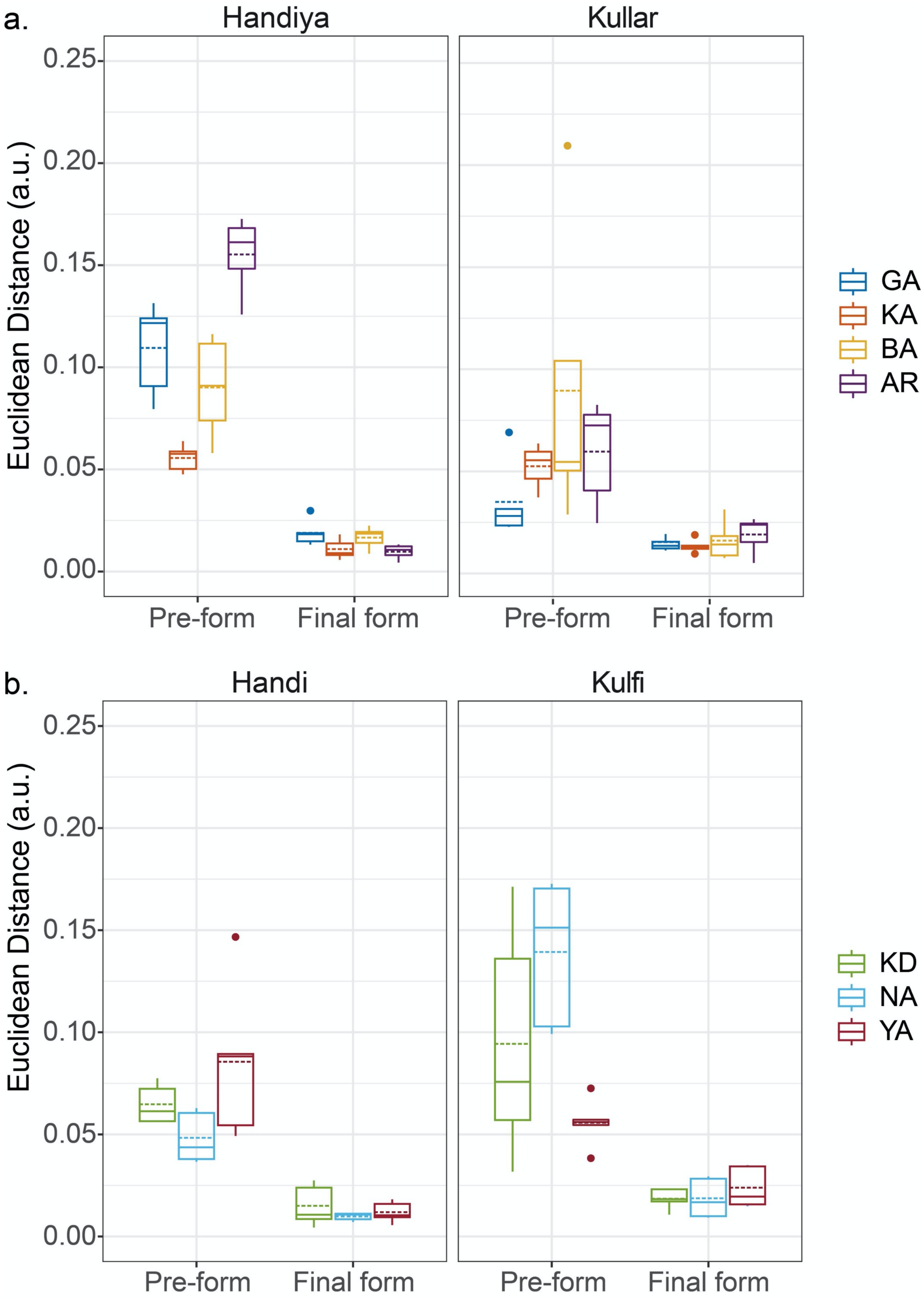

Supplement: S4 Fig — (PDF) [file pone.0239362.s004.pdf]
